# From union clout to corporate couture: Unveiling the impact of trade unions on corporate social responsibility

**DOI:** 10.1371/journal.pone.0311244

**Published:** 2025-01-09

**Authors:** Anshul Mandliya, Jatin Pandey, Shrihari Suresh Sohani, Rayees Ahmad Sheikh

**Affiliations:** 1 Organizational Behaviour and Human Resource Management, Goa Institute of Management, Sattari, Goa, India; 2 Organizational Behaviour and Human Resource Management Area, Indian Institute of Management Indore, Indore, Madhya Pradesh, India; 3 Economics Area, Indian Institute of Management Indore, Indore, Madhya Pradesh, India; Pontifical Catholic University of Rio de Janeiro: Pontificia Universidade Catolica do Rio de Janeiro, BRAZIL

## Abstract

Trade unions and corporate social responsibility (CSR) are important institutional mechanisms through which corporations undertake responsibility for their for-profit as well as not-for-profit actions. This study explores the roles of trade unions, management, and shareholders in influencing environmental, social, and governance (ESG) controversies and CSR reporting. Drawing on instrumental stakeholder theory, we argue that while management and shareholders typically focus on governance, profitability, and operational efficiency, trade unions act as critical relational stakeholders advocating for employee welfare and social sustainability. Using a sample of 416 firms over a period of 10 years, our findings reveal that trade unions significantly reduce ESG controversies and enhance CSR reporting by advocating for employee welfare and social sustainability. In contrast, management significantly impacts CSR reporting but notably does not influence ESG controversies. Lastly, shareholders, who are important for governance and profitability, exhibit minimal impact on both ESG controversies and CSR reporting.

## Introduction

Corporate social responsibility plays an important role in determining the firm’s reputation and market value across different stakeholders [1–3]. Stakeholder theory emphasizes that firms are responsible to multiple stakeholders to achieve long-term success and legitimacy [4]. Due to the growing pressure from lawmakers, government bodies, investors, and society, these stakeholders are becoming more prominent from the perspective of environmental and social sustainability [5]. These firms which give importance to them further enjoy support from multiple other stakeholders such as investors, policy-makers, media houses, and rating agencies [1, 6].

In recent times, the sustainability of firms along with its CSR performance is seen to be measured from multiple metrics. One such metrics is Environmental, social, and governance (ESG) performance scores [7] of the firm. ESG scores have gained popularity across a range of internal and external stakeholders. Many investors, consumers, and law firms have started recognizing the importance of ESG scores and have openly expressed their support in pressing the organizations to release the same [8, 9]. These scores play a key role in disciplining the firm’s behaviour and action towards issues of environmental protection, human rights, and CSR disclosures [10].

One of the important stakeholders in CSR activities are employees, as they play a key role in implementing and executing sustainable goals at the ground level. The employees have a dominant position in communicating the social performance of the organisation which includes its workforce satisfaction, and human rights activities [11]. However, these employees who tend to expose the irregularities in the ESG performance of the organization might face a series of discontentment from the management. It becomes important to support the voices of these individuals for the desirable and ethical functioning of a firm in the market. Thus, trade unions become an essential stakeholder in the organization as it provides a protective voicing and negotiation platform for its members to express their concerns related to working conditions, compensation, and treatment of workforce within the organization [12].

The extant literature in CSR, corporate social performance [13], and corporate social irresponsibility [6] have not paid much attention to employees and their voice in the form of trade unions (TU) [14]. The attention has been predominately given to sustainable and responsible investment [15], media coverage of corporate scandals and controversies [16–18], corporate governance [19, 20] and CSR disclosures [20]. The current study is aimed at understanding the unique relationship between CSR and trade unions as these mechanisms were not given equal focus by the organizations at the same time. When scholars and practitioners were focusing on industrial relations and trade unions, the area of corporate social responsibility was emerging, and when CSR activities became predominant across the world, the unions have started declining.

Based on the review of extant literature, two research gaps emerge: the first pertains to the role of trade unions as a stakeholder in reducing ESG controversies [21]. Management and shareholders have different motives to influence corporate strategies, whereas trade unions can prioritize employee-oriented and non-employee-oriented CSR [14]. Thus, in comparison to management and shareholders, trade unions’ may be better in a position to identify ethical concerns to enable corrective measures which in turn have potential to reduce corporate social irresponsibilities (CSiR). In other, words, research needs to evaluate whether due to their unique position, trade unions’ involvement may reduce ESG controversies when compared with management and shareholders.

The second gap in literature pertains to CSR disclosures. While scholars have studied the role of board diversity [20, 22] and management practices [23] on CSR reporting, the role of trade unions remains under examined [12]. Since trade unions may have bargaining power to hold organizations accountable for employee rights, labour practices and social impacts [24], involving them in CSR disclosures may impact transparency and clarity of disclosures. In other words, it is imperative to understand how trade unions’ involvement may impact CSR disclosures.

This study aims to address the research gaps by examining the role of trade unions as stakeholders in CSR. For doing so, the paper empirically compares the impact of trade union representation vis-à-vis management and shareholders on 1) reducing ESG controversies, and 2) enhancing CSR reporting. The paper seeks to understand how participation of trade unions can contribute to social sustainability of organizations through influence on CSR.

The study contributes to the existing literature on corporate sustainability and industrial relations in three important ways. First, it sheds light on the distinctive role of trade unions in CSR, using stakeholder theory to emphasize their importance as key stakeholders [25]. This approach highlights trade unions as vital stakeholders, reinforcing the idea that addressing diverse stakeholder interests is crucial for organizational success and legitimacy [4, 25]. Second, it offers a comparative analysis of the impact of trade unions versus management and shareholders on ESG controversies and CSR reporting. This comparison provides valuable insights into how different stakeholders affect CSR outcomes, which can help both practitioners and researchers in effectively focusing their interest. Third, the study addresses the broader issue of the declining influence of trade unions across various organizational contexts. By presenting evidence of ongoing significance of trade unions in CSR and sustainability, the study fills a critical gap in the literature and underscores the need to recognize the contributions of trade unions in ethical and sustainable business practices [26].

## Literature review and theoretical framework

The existing literature on CSR is extensive. To better contextualize the research questions, this study organizes the broad literature on CSR and trade unions under the following headings:

### Environmental, social and governance (ESG) measures

Performing good on CSR measures is becoming important for organizations to establish a positive image in society and market, when incidences of environmental, social, and governance scandals and controversies are emerging all over the world [27]. The ESG scores provide a comprehensive metrics to study the important facets of CSR-sustainability across industrial sectors and organizations [28, 29]. The firms and their investors view these ESG scores from different perspectives. For firm’s top management, ESG scores provide a source of legitimacy across its external stakeholders and a competitive advantage against its rivals. While, investors use it in gauging the long term sustainability of the firm, as investments in socially responsible firms tend to give higher financial returns in the market [22, 30]. Overall, CSR score provide an indicator of performance for enhancing the financial and social sustainability of the firm [31].

Additionally, CSR measures also help an organization measure and compare their corporate social performance. Thereby giving organization a new perspective to evaluate and modify its strategy as per the demands of its stakeholders [32]. In the last few decades, concerns towards sustainability have increased multifold, which has directed the focus of a substantial number of stakeholders towards the firm such as consumers, government agencies, lawmakers, and activists [33]. These stakeholders along with media houses play a significant role in communicating the social image of the organization which has substantial impact on its financial performance. An organization with an irresponsible image might invite a series of negative responses from its investors, shareholders, and potential employees [6]. However, an organization with a socially responsible image not only receives support from its external stakeholders but have higher employee identification and commitment as such organizations can enhance the social image of its employees [34, 35].

The three major dimension of ESG score focus on certain specific attributes of the firm. The environmental dimension focuses on emissions and waste disposal, resource uses and renewable energy investments, and environmental innovations and designs. The governance dimension focuses on CSR reporting and strategy, management compensation and representation (diversity, independence), and shareholder rights. The social dimension focuses on business ethics and community involvement, human rights and labour laws, workforce satisfaction and equal representation, and product responsibility [36]. Firm’s aim to improve their performance along these three dimensions and their respective sub-dimensions to enhance their social image [37], and boost financial performance & market value [7]. ESG scores thus provide a key metric for evaluating CSR performance and its impact from various stakeholders. To understand this impact better, the study examines how CSR performance relates to three major stakeholders: employees or trade unions, management, and shareholders. Stakeholder theory serves as a theoretical framework for examining the dynamics between various stakeholders and CSR performance.

### Trade unions

With the increase in industrialization in the late 19^th^ and early 20^th^ century, many migrant workers, miners and other workmen started moving to industrial areas within the continental Europe and Americas [38]. These workers started forming alliances which provided them a platform for negotiation and voicing their concerns with the senior management [39]. After the World War the socialism and unionism saw rise in industrial countries, where working class became a prominent source of political support [38]. The unions voiced workers’ demand for health and safety, conflict resolution, job security and any other concern related to workers’ treatment within the firm [40].

Worker’s concern is an important factor within the social dimension of sustainability [41]. This makes working standards a vital part for achieving the sustainable goals of inclusivity, responsiveness, and representation. The collective bargaining potential of labour unions provides distributive justice in the form of compensation and workplace treatment [42]. Along with this, trade unions provide voice and a vigilance mechanism to monitor organizational activities. Organizations around the world have started adopting co-determination practice, where worker representatives get the right to become part of the board of directors of the company [43]. Co-determination provides the ground-level voices to the senior management thus supports a bottom-up approach in innovation and strategy formulation [43].

### Stakeholder theory

Organizations are surrounded by a multitude of stakeholders [44]. As per Freeman (1984), these stakeholders are interconnected with each other and collaboratively produce value for the organization and themselves. The stakeholder theory emphasizes that an organization must aim to create value for all of its stakeholders instead of just its shareholders [4, 44]. It intermixes the themes of business and ethicality to include the doctrine of fairness, feminist theories, ecological preservation, and other similar standpoints to create value for different stakeholders associated with the organization [25, 45, 46]. There are three facets of stakeholder theory: descriptive, instrumental, and normative [44]. The descriptive facet focuses on describing the organization as a set of cooperative and competing values that helps us in understanding how the organization affects multiple stakeholders and the wider environment around it. The instrumental facet focuses on defining and achieving instrumental goals such as profits, growth, and sustainability. The normative facet focuses on stakeholders as entities (person or group) with legitimate interest in the organization that create intrinsic value for the specific stakeholder [44, 47].

Stakeholder theory emphasizes how value is created through the relationships an organization has with various actors and how these actors can influence or be influenced by the organization [25, 48]. Stakeholder thinking introduces new ways of looking at the firm beyond their functional objectives of shareholder wealth maximization. It questions the process of value creation and likewise trade-offs which an organization undertakes to achieve its goals [46]. Stakeholder theory just like corporate social responsibility (CSR) focuses on the wider obligations of the firm towards society. Although, CSR only talks about the financial consideration of the firm towards such obligation, stakeholder theory conceptualizes a much larger array of considerations which include social and environmental factors [44, 46, 48]. From the stakeholder theory perspective, CSR directs the focus of the organisation towards its labour practices and environmental efforts [49]. Stakeholder theory emphasizes that stakeholders are interdependent and activities that create or support one stakeholder creates value for others [50]. In addition to this, sophisticated manufacturing industries do better when they adopt stakeholder-oriented systems [51, 52].

These stakeholders can influence firm’s behaviour towards increasing its environmental and social sustainability [53–55]. They can demand for or regulate a firm, enhancing its social performance, while decreasing its negative impact towards the society and the planet. The recent spike in corporate scandals and firm’s unethical behaviours toward society and environment calls for the inclusion of multiple stakeholders and their interests in the firm’s value creation activities [56]. One such stakeholder is trade union which has been overlooked from major CSR-related discussion. The following section highlights the importance of integrating employee relations perspectives into stakeholder theory and CSR literature [14].

### Stakeholder view in employee relations literature

Employees are an important stakeholder of the firm, however the literature on stakeholder theory has rarely shown normative concerns about their employment relationships [57]. The industrial relations literature has emphasized on trade unions and workers ill treatment within the firm but has rarely discussed TUs from the perspective of stakeholder theory [57, 58]. Stakeholder theory discusses that identification and salience of the stakeholder can be based on one or more of the three attributes they possess: power, legitimacy, and urgency [59]. In the context of CSR and social sustainability, these attributes can help us identify salient stakeholders which can improve the firm’s social performance [25, 59]. Employee relations can be a primary stakeholder in CSR and social sustainability of the organization as covered in corporate social performance literature and ratings such as KLD [60].

The cross-fertilization of Employ Relations (ER) and stakeholder theory literature can inform us that enhancing stakeholder voice and representation in CSR conversation may positively contribute to a holistic value creation by the firm which can give us a deeper understanding of the stakeholder theory [57]. ER scholars offer a more pluralistic understanding of the organization and highlight the influence of trade unions in effectively enhancing the social purpose of the organization [61]. Through an ER lens, trade unions can be a significant collaborator in CSR as they are directly connected to the ground level activities as well as are protected by bigger institutional frameworks and legislation. From a stakeholder theory point of view, trade unions can be an important relational stakeholder of the firm due to their communal sharing relational ethics (CSRE) approach [62]. Trade unions can be an effective contributor in CSR deliberation of the firm as they possess bargaining, monitoring, coordinating, and coercive capabilities in the firm [12]. The further section provides an in-depth understanding on trade unions and how they can impact the CSR performance of the organization.

### Hypotheses development

#### Stakeholders of the firm

There are three major stakeholders of an organization: shareholders & board of directors, management or executives, and employees [63, 64]. The literature in corporate governance recognizes shareholders and management as dominant stakeholders. Shareholders, as owners, hold authority over the recruitment and evaluation of senior executives, while management controls the day-to-day operations and policy implementation [65]. Literature on social responsibility of the organization has also included employees’ voice and justice as important parameters of social performance [64]. The trade unions are an important stakeholder along with shareholders and management within the organization to improve its corporate social image. The trade unions possess the capability to bargain their rights with the management. The management focuses on adhering to the best practices of corporate governance which includes monitoring and improving policy and audit boards in the organization [66]. Shareholders’ focus on equal rights, diverse representation and other measure which could compromise the best standards of practice within the organization [67]. Having understood the importance of relationship between CSR and trade unions from the perspective of stakeholder theory, we discuss organizational controversies are impacted by these stakeholders.

#### Organizational controversies

Organizational controversies involve incidents of environmental, social, and governance (ESG) irresponsibility by the organization. These controversies may include actions such as concealing environmental damage, manipulating reports on green investments, discriminating against employees or board members, and other similar activities that attract negative attention from the media and the public [18, 21]. These events can damage the image of the organization across its stakeholders. In serious cases, the organization might attract penalties and sanctions from law and governing bodies. These events can also bring shame and dissatisfaction among the internal stakeholders of the firm as they tend to distort the construed positive image of the organization [37]. ESG controversies are seen in the extant literature to affect the financial performance and market value of the organization [68, 69].

The source of organizational controversies is believed to be present within the organization which includes activities of social and environmental ignorance, wage disputes, discriminative practices, employee health and safety controversies, human rights controversies, and other frauds of consumer and public interest [18]. The three dominant stakeholders within the organization can act as a deterrent to such events of irregularities within the organization and thus possess the potential to avoid such controversies from happening.

Trade unions are key social stakeholders in organizations, as their deep-rooted social and welfare-oriented interests directly impact the organization’s social performance [24, 47]. Trade unions interests are tied with civil society on the matters of corporate social responsibility for social development [24]. By acting as internal watchdogs and mediators between firms and government regulations, trade unions can help enforce compliance with labour laws and ethical practices, thus minimizing the likelihood of legal or governance-related controversies. Additionally, unions’ focus on transparency and accountability increases internal scrutiny, encouraging firms to avoid practices that could lead to negative public attention or media backlash. Consequently, increased trade union representation can reduce the likelihood of occurrence of organizational controversies, as their involvement drives firms toward more ethical and socially responsible behaviours.

From the stakeholder theory perspective, management serves as a critical operational stakeholder, directly influencing the firm’s internal practices and external stakeholder relationships [4, 70]. Effective management is essential for ensuring that ethical standards are upheld, and that regulatory compliance is rigorously maintained. When management demonstrates high effectiveness, it means they are proficient in implementing controls, fostering transparency, and addressing potential risks related to environmental, social, and governance (ESG) issues [47]. Effective management can thus mitigate the likelihood of ESG controversies by proactively addressing and managing these risks. Therefore, firms with higher levels of management effectiveness are expected to experience fewer ESG controversies due to better internal controls and a stronger commitment to ethical and sustainable practices.

The shareholders of the firm have one of the biggest stakes to lose in terms of their wealth when it comes to controversies and frauds. This directs their behaviour to be more proactive towards corporate governance and monitoring of the firm’s operational and financial activities [16]. As a significant financial stakeholder of the organization with direct interest in organization’s financial performance and profitability, shareholder’s effectiveness in terms of corporate governance will be negatively associated with occurrence of organizational controversies.

Based on the above discussion, we can propose the following hypothesis:

*H1*: *At higher levels of trade union representation*, *firms will have fewer ESG controversies*.
*H2*: *At higher levels of management effectiveness*, *firms will have fewer ESG controversies*.
*H3*: *At higher levels of shareholder effectiveness*, *firms will have fewer ESG controversies*.

#### CSR reporting

In contemporary contexts, corporations occupy a pivotal position within both the economic and societal spheres. They are expected to uphold uncompromising integrity in their business practices and societal responsibilities, including, but not limited to, fair trade and the protection of human rights for their direct and indirect beneficiaries. Their impact on society extends beyond merely fulfilling legal and regulatory requirements, encompassing broader social and environmental responsibilities [71]. CSR reporting becomes a crucial tool in this regard, as it enables organizations to transparently disclose their efforts in addressing social and environmental concerns [72]. Through such reporting, firms can communicate their commitment to ethical standards, demonstrating accountability to both internal and external stakeholders [73].

Profit is viewed as a natural outcome of the organization and in capitalist societies, a prime component to assess the success of the firm, which directly affects its relationship with its shareholders and investors. The management is the medium through which organization achieves its profit goals and employees are a direct contributor to the activities through which firms make their profits [74, 75]. For all these three stakeholders CSR reporting becomes an important parameter to reflect on their organization’s performance.

Trade unions are an important social stakeholder of the firm as the firms’ policies can directly impact their work conditions and livelihood. They are protected by protected both intra-national and international entities [12]. Trade unions can also engage in activities of CSR deliberation and reporting as they help in monitoring, regulating and implementing best practices of social and environmental sustainability [53]. With the co-determination practice, trade unions can have significant involvement in the CSR strategy and reporting which is observed to improve the CSR-related activities in the firm such as adoption of emission reduction targets, disclosure of CSR reports, and responsibility towards employment security [43]. Thus, higher trade union representation can improve organization’s CSR reporting as they have direct interest in organization’s social performance.

Management, as operational stakeholders, plays a pivotal role in implementing corporate policies, including those related to CSR [4]. Since CSR efforts directly impact a firm’s social image and reputation, management often aims to enhance these efforts through transparent reporting and proactive engagement in social and environmental sustainability initiatives [9]. As organizations face increasing scrutiny from external stakeholders, effective management works to improve CSR practices and disclosures to avoid reputational damage and foster a positive public image [6]. High-quality CSR disclosures enhance public assurance and strengthen the firm’s reputation by demonstrating commitment to social and environmental standards [76]. Thus, effective management can improve CSR reporting for reinforcing the firm’s social and environmental credibility.

Shareholders, initially focused on profitability, are now also concerned with social and environmental performance due to its impact on long-term sustainability and reputation [77]. Institutional investors are particularly influential in this shift, pushing for greater CSR transparency and suggesting ways to improve CSR ratings [78]. In the wake of corporate social irresponsibility (CSiR) events, shareholders increasingly show interest for eco-friendly initiatives, rewarding companies with positive environmental practices and penalizing those engaged in eco-harmful activities [79, 80]. This shift indicates that shareholders recognize how CSR contributes to firm value and reputation. [77]. As a result, they actively advocate for enhanced CSR practices and transparency [79]. Thus, firms with higher shareholder effectiveness are more likely to demonstrate better CSR reporting, as they are motivated to show accountability and attract long-term investment.

Based on the above discussion, we can propose the following hypothesis:

*H4*: *At higher levels of trade union representation*, *firm will have better CSR reporting*.
*H5*: *At higher levels of management effectiveness*, *firm will have better CSR reporting*.
*H6*: *At higher levels of shareholder effectiveness*, *firm will have better CSR reporting*.

## Methodology

### Data sample

The data for the study is collected from Thomson Reuters ESG database (currently, LSEG) available under Refinitiv Terminal. The database has more than 140000 firms spreading across different industries for more than 400 ESG metrics and indicators to evaluate their performance on environmental, social and governance dimensions [36, 81]. For this study, we focused on firms in manufacturing industry (Energy, Basic materials, Cyclical products, Non-cyclical products, Industrials, Healthcare, utilities and Technology) as they tend to have a higher impact on environmental and social sustainability in the form of environmental pollution, waste disposal, green products and renewable energy compared to the service sector firms [82]. Also, the presence of unions and union affiliations tend to be higher in the manufacturing sector than in the service sector [83].

The companies have been selected based on the presence of their Trade union representation scores on the Thomson Reuters ESG panel [31], which is the focal variable of this study. A set of 2200 companies were initially identified based on the above mentioned criteria and the data for these firms is retrieved for a period of 10 years (2009 to 2019) [84]. Furthermore, we selected the firms for which data is available for all the variables for all the time periods in consideration, that left us with 416 firms. The intent is to run the regression on fully balanced panel of 416 firms. But, for brevity we have used a partially unbalanced panel where data for all firm for all variables is available for at least 8 years increasing the number of firms to 676. The summary of descriptive and regression results for partially unbalanced panel of 676 firms and fully unbalanced panel of 2200 firms is presented in S2 Appendix. The distribution of these 416 firms across eight sectors in manufacturing industry and across different institutional categories are available in Tables 1 and 2, respectively. In addition to trade union score, we also captured management score, shareholders score, ESG controversy score, and CSR Sustainability Reporting Score and total revenues. The definition of these constructs from the Thomson Reuters Eikon terminal is placed in S1 Appendix.

**Table 1:** Distribution of firms across different manufacturing sectors (N = 416).

Industry	Number of entries	Percentage
Energy	460	11.06
Basic Materials	740	17.79
Industrials	730	17.55
Consumer cyclicals	660	15.87
Consumer non-cyclicals	310	7.45
Healthcare	160	3.85
Technology	560	13.46
Utilities	540	12.98
Total	4,160	100

**Table 2:** Distribution of firms across different institutional categories.

Institutional Category	Number of entries	Percentage
Collaborative Agglomerations	40	0.97
Coordinated Market Economy	700	16.99
Centralized tribe	0	0
Emergent Liberal Market Economy	190	4.61
Family led	220	5.34
Hierarchically Coordinated	260	6.31
Liberal Market Economy	2,470	59.95
State Led	240	5.83
Total	4,120	100

Note: Missing information on 40 points in institutional categories are classified as others

### Variable selection

Dependent variable—The dependent variables for the study are ESG controversies and CSR reporting. Both constructs are important from economic and social perspectives as they help in regulating and standardizing best practices of fair trade and sustainability. Thomson Reuters Eikon database [31] follows GRI framework for reporting CSR practices which are then converted to a finalized score [85]. Further details about these scores are covered in S1 Appendix. For ESG controversy score, Eikon database [31] focuses on the controversies which happened across the three dimensions of environment, social, and governance categories and their 10 subcomponents in the last fiscal year, the detailed method have been referred in the previous literature [18]. The details for the same is available in S2 Appendix.

Independent variables—The Independent variables for the study are trade union representation score, management score, and shareholder score [31]. The two category scores shareholder scores and management scores are calculated by Thomson Reuters ESG database by counting the specific points for best practices under each category and then giving a percentile rank score. The formula for the same is as follows [18, 31].


$$score=\frac{no.of\;companies\;with\;a\;worse\;score+\frac{no.of\;companies\;with\;the\;same\;value\;included\;in\;current\;one}{2}}{no.of\;companies\;with\;a\;value}$$

Data for trade union representation score is collected from Thomson Reuters Eikon terminal, similar data has been used in previous research as a measure union density within the organization [14, 86]. Detailed mythology adopted by Refinitiv for calculating the trade union representation score [31] is available in S1 Appendix.

Control variable- The control variable is Log of Total Revenue of the firm. This variables has been used in previous research [14].

### Empirical estimation and data analysis

Panel data regression modelling approach is used for analysing the effects of trade union representation, management, and shareholder on ESG controversies and CSR reporting. Based on the suitability of the data analysis technique, econometric modelling is used to test the proposed relationships The data analysis is carried out with Stata 16.1. In order to establish the relationship between outcome and explanatory variable of our interest we have use fixed effect Panel Data Regression model as follows:

$${ESG\;Controversy}_{it}=\beta_{1}+\beta_{2}{TUR}_{it}+\beta_{3}{MS}_{it}+\beta_{4}{SS}_{it}+e_{it}$$ (1)

$${CSR\;Reporting}_{it}=\beta_{1}+\beta_{2}{TUR}_{it}+\beta_{3}{MS}_{it}+\beta_{4}{SS}_{it}+e_{it}$$ (2)

Where *ESG Controversy*_*it*_ (Environmental, Social, and Governance) and *CSR Reporting*_*it*_ (Corporate Social Responsibility) are the outcome variables interest for firm “*i*” at time “*t*” TUR, MS and SS represent trade union score, management score, and shareholder score are the explanatory variables of interest for firm “*i*” at time “*t*”. *β*_1_, *β*_2_, *β*_3_, *and β*_4_ are the parameters of equation and *e*_*it*_ is error term assumed to follow normal distribution. First, we checked for panel data, the presence of cross-sectional dependence. Using Pesaran CD test [87], we found there is no evidence for cross-sectional dependence in data. Next, we used Levin–Lin–Chu test for the presence of unit root test in data [88]. The test results suggested that unit root is not a concern in our data. Further, Hausman tests [89] was conducted for each of the hypotheses to choose the appropriate model among fixed and random effects. The results suggested that Panel fixed effect model is best fit in our case. We control for the year dummy to account for time fixed effects in addition to entity fixed effects. In addition to this, we have accounted for country, industry, and varieties of institutional systems [90] in our fixed effect models.

## Results

We have run all the tests for three sets of panels viz. fully balanced panel, unbalanced panel, partially balanced (panel with all variables having information on 8 or more years) and partially balanced panel with imputed values. We have discussed a fully balanced panel here and appended the rest of the results for the sake of brevity in S2 Appendix.

The descriptive statistics of the variables are placed at Table 3. Table 4 contains the bivariate correlation between the variables of the study. Table 5 shows the main results of regression. Model 1 is indicative of the relationship between these variables of interest without the presence of control variable (Log of total revenue). Model 2 presents the results with control variable. In model 3, we have accounted for country, industry, and varieties of institutional contexts [90] for addressing differences in labour policies and markets. The results in Table 5 report that trade unions have a significant negative effect on ESG controversies, which supports hypothesis 1. Results from Table 5 also reports that management and shareholders have no significant effect on ESG controversies, which rejects hypothesis 2 and 3.

**Table 3:** Summary statistics (N = 416).

Variable	Mean	Std. Dev.	Min.	Max.
Trade Union Representation	42.772	33.57436	0	100
Management Score	60.81254	26.37355	1.041667	99.98057
Shareholder Score	53.41346	28.79451	0.139276	99.8496
Log of Total Revenue	22.5133	1.26622	16.02856	26.88825
Total Revenue	13700000000	29100000000	9143581	476000000000
ESG Controversy Score	84.87113	27.39508	0.704225	100
CSR Reporting Score	55.13682	34.13535	0	99.60317

**Table 4:** Bivariate correlation between variables (N = 416).

Variables	(1)	(2)	(3)	(4)	(5)	(6)	(7)
(1) Trade Union Representation	1.000						
(2) Management Score	0.017	1.000					
(3) Shareholder Score	0.013	0.098***	1.000				
(4) Log of Total Revenue	0.249***	0.211***	0.020	1.000			
(5) Total Revenue	0.199***	0.122***	-0.034**	0.636***	1.000		
(6) ESG Controversy Score	-0.100***	-0.064***	-0.024	-0.333***	-0.304***	1.000	
(7) CSR Reporting Score	0.505***	0.183***	0.063***	0.398***	0.239***	-0.192***	1.000

Note. *p < 0.1

**p < 0.05

***p < 0.01

**Table 5:** Results of regression analysis for ESG controversy (Sample of 416 firms for 10 years).

	Model 1	Model 2	Model 3
VARIABLES	Fixed Effect	Fixed Effect with control	Fixed Effect with country, industry, and category controlled (134 combinations)
*Independent Variables*			
Trade union Representation	-0.193***	-0.198***	-0.187***
	(0.054)	(0.054)	(0.022)
Management score	-0.008	-0.006	-0.003
	(0.021)	(0.021)	(0.017)
Shareholder score	-0.020	-0.020	0.006
	(0.021)	(0.021)	(0.015)
Year Effects	Yes	Yes	Yes
*Control Variable*			
Log of Total Revenue		-1.929	-6.644***
		(1.416)	(0.429)
Constant	89.066***	132.805***	237.435***
	(3.086)	(32.262)	(9.408)
Observations	4,160	4,160	4,120
R-squared	0.027	0.027	0.254
Number of identifiers	416	416	416

Note. *p < 0.1, **p < 0.05

***p < 0.01. Standard errors in parentheses.

**Fig 1:**
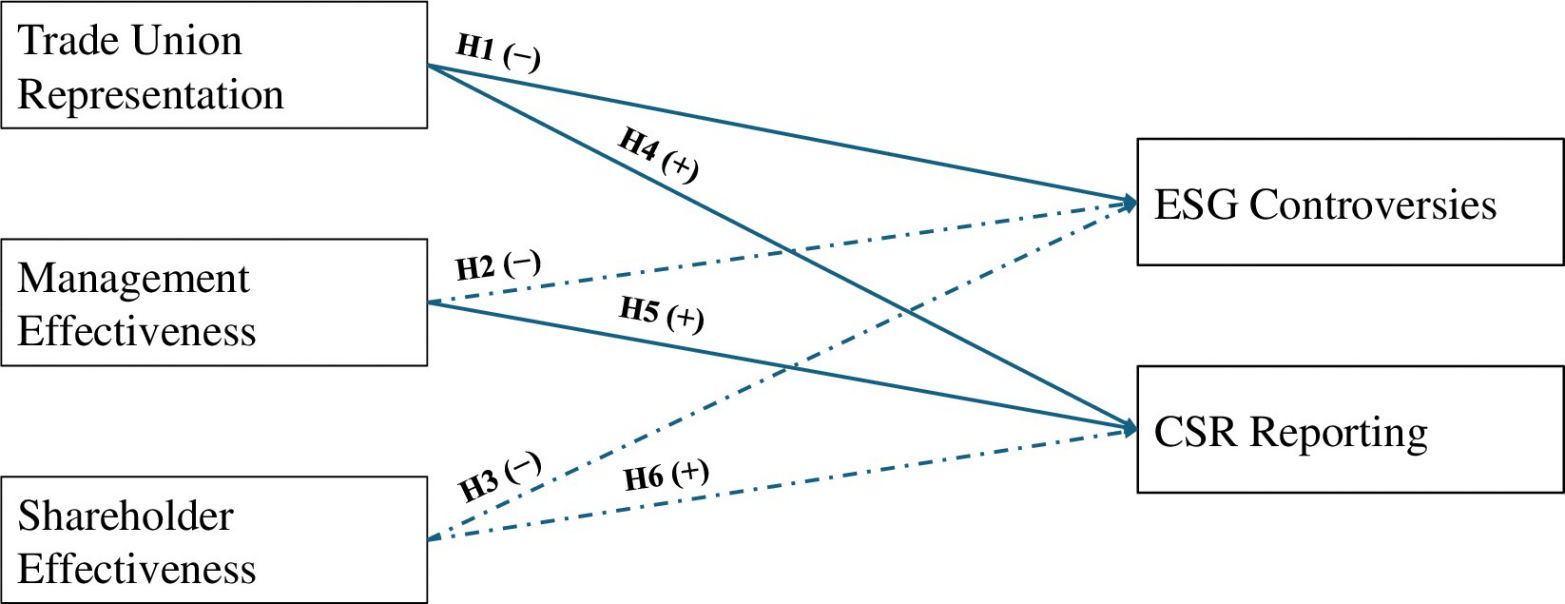
Hypothesis testing results.

Table 6 shows the relationship between independent variables and CSR reporting. Model 1 reports that trade union representation has a significant positive effect on CSR reporting; the same effect is sustained in the presence of control variable in model 2, which supports hypothesis 4. The effect of management and shareholders on CSR reporting is also found to be significant and positive in both model 1 and 2 which supports hypotheses 5 and 6. However, in model 3, where we accounted for country, industry, and institutional category effects, only trade unions and management shows positive significant effects on CSR reporting, while shareholder’s do not show any significant effect on CSR reporting. The final results from hypothesis testing are available in Fig 1.

**Table 6:** Results of regression analysis for CSR reporting (Sample of 416 firms for 10 years).

	Model 1	Model 2	Model 3
VARIABLES	Fixed Effect	Fixed Effect with control	Fixed Effect with country, industry, and category controlled (134 combinations)
*Independent Variables*			
Trade union Representation	0.091***	0.097***	0.342***
	(0.032)	(0.032)	(0.021)
Management score	0.065***	0.063***	0.118***
	(0.012)	(0.012)	(0.016)
Shareholder score	0.021*	0.021*	-0.002
	(0.013)	(0.013)	(0.014)
Year Effects	Yes	Yes	Yes
*Control Variable*			
Log of Total Revenue		1.994**	9.123***
		(0.842)	(0.408)
Constant	53.345***	8.130	-165.961***
	(1.835)	(19.177)	(8.946)
Observations	4,160	4,160	4,120
R-squared	0.081	0.082	0.561
Number of identifiers	416	416	416

Note. *p < 0.1

**p < 0.05

***p < 0.01. Standard errors in parentheses.

Overall, for the fully balanced panel, we observed trade unions have a significant negative effect on ESG controversies and a significant positive effect on CSR reporting. The effect of shareholders became insignificant when we accounted for country, industry, and institutional category effects. We observed similar results with unbalanced panel, partially balanced panel (panel with variable having information on 8 or more years), and partially balanced panel with imputed data.

## Discussion

Trade unions, management, and shareholders are important stakeholders in an organization with varied stakeholder interests. Shareholders, as financial stakeholders, are primarily driven by the goal of maximizing returns on their investments. In contrast, management, focusing on operational efficiency, seeks to boost organizational profit by optimizing resource use. Meanwhile, trade unions play a crucial social role, championing employees’ interests through collective bargaining and safeguarding them from exploitative practices. This interplay highlights a divergence in priorities: while shareholders and management are concentrated on financial and operational outcomes, trade unions address social equity and social outcomes.

As employees in trade unions are supported by larger institutional structures both within and outside the firm [91], they are better positioned to identify and report any irregularities occurring at the ground level [12, 38]. Trade unions enhance workers’ treatment and help report organizational irregularities, which reduces the risk of ESG controversies. In contrast, shareholders and management, focuses on financial gains, wealth maximization, and operational efficiency [16, 92, 93], may overlook ESG controversies due to their distance from day-to-day employee activities. Our results demonstrate that higher trade union representation is associated with reduced ESG controversies, while management and shareholders did not show any significant effect. This suggests that, although management and shareholders may neglect ESG concerns due to their focus on financial performance, trade unions are vital in addressing these risks through their focus on social equity and worker protection.

Instrumental stakeholder theory suggests that organizations achieve positive performance consequences when they maintain ethical and fair relationships with their stakeholders [49]. Employees are classified as primary stakeholders for an organization as they create value by performing activities along with management. However, shareholders are also considered as primary stakeholders as they provide important resources such as capital to the organization [55]. Behavioural economists identified individual motives for fairness in two categories: self-regarding, one who are inclined to focus on increasing their payoffs, and the reciprocators, who focus on increasing the payoffs for both society and self [49]. Considering this arguments on firm’s value creation, reciprocal stakeholders such as management and trade unions have higher likelihood on increasing firm’s value creation when the firm adopts a fairness approach such as CSR [49]. This is demonstrated with the significant positive relationship observed between trade unions and management with CSR reporting. However, self-regarding stakeholders such as shareholders can only add to firm’s value creation when it adopts an arm-length approach [49]. This suggest that investors due to their higher transactional orientation and beliefs in monetary gains may not contribute much to value creation when firm’s chooses fairness approach such as CSR reporting. This can be seen in the insignificant or less significant relationship of shareholders with CSR reporting. Further, trade unions and management are also willing to support CSR reporting in their organisation; trade unions for achieving better work conditions and wages [12], while management to enhance the social image and reputation of the organization [37].

Lastly, in the wake of events of corporate social irresponsibility such as Enron, Volkswagen and other similar scandals, it becomes important to involve other stakeholders of corporate social responsibility which can keep an eye on the corporate governance practices within the firm [53]. Board diversity may also enhance responsibility concerns of a firm [94]. Groups such as trade unions are recognized by law in major countries across the world and have an external affiliation to other stakeholders who can prevent CSR controversies and scandals from happening [12]. The result of this study shows that trade union representation reduces the ESG controversies and improves CSR reporting of the firm.

### Theoretical and practical contributions

Our results demonstrate that trade unions are significant social stakeholders in the firm from the perspective of stakeholder theory. In comparison to the management and shareholders who have more instrumental interest in the firm, trade unions focus on building and sustaining relational interests with the firm by looking after employee welfare and social sustainability. This supports the relational view of stakeholder theory, which argued for treating stakeholders as "ends" rather than mere "means" [95]. Trade unions, which promote communal sharing relational ethics (CSRE) by forming close, cooperative relationships with the organization and its employees can be helpful in developing firm’s strategy by keeping in mind the social purpose and sustainability obligations of the firm [62]. This CSRE approach can benefit firms in enhancing their cooperation and reducing costs of organizational controversies and other CSR-related issues [62]. Moreover, this advocacy supports the notion that firms "do well by doing good" and not only focuses on enhancing their profits and operational efficiency [62].

This study adds to the current literature on stakeholder theory [4, 62] by positioning trade unions as an important relational stakeholder [95] that can reduce organizational controversies and improves CSR reporting. It also embraces the notion that management and shareholders are significant drivers of CSR strategy, however, when it comes to grassroot-level implementation and monitoring, they may not be well equipped to ensure CSR and sustainability goals are met by the firm [12]. Thus, it becomes important to involve relational stakeholders such as trade unions not only at the macro institutional representations but also in micro-organizational processes such as CSR implementation.

For practitioners, the research highlights the need to build strong trade union relationships, not only for compliance measures but to advance and monitor their CSR strategy and practices. Including trade unions in CSR strategy can be helpful for firms towards maintaining effectiveness and transparency of CSR at the ground levels [53]. This can enhance organizational credibility and reduce the risk of ESG controversies and irresponsibilities. Ultimately, for shareholders and management, trade unions can be a great resource in learning internal and external conversations about sustainability and CSR issues.

## Conclusion

Studies on CSR and social performance have predominantly focused on management [29, 96, 97] and shareholders [98–100] while ignoring other stakeholders such as trade unions. Trade unions, representing the collective voice of employees, can direct firms’ attention to social and ethical issues. However, a decline in the presence and involvement of trade unions has been noted across firms [24, 101]. This study explores the under-researched area of how trade union representation affects ESG controversies and CSR reporting. These indicators are integral to broader environmental, social, and governance (ESG) scores, which have increasingly attracted the attention of socially responsible investors [15], rating agencies, and media outlets [10, 17], and both current and potential employees of the firm [102].

Organizations across the global are investing heavily in ESG practices to align themselves with sustainable goals, to have an environmentally and socially sustainable image which could help them boost their financial growth and market reputation. However, they have been failing to achieve the same as the management and shareholders tend to have limited visibility of ground level operations and their direct interest falling in the zone of wealth maximization [74, 103] leaving corporate social irresponsibility to emerge [6].

According to the study findings, trade unions are emerging as crucial stakeholders in improving CSR disclosures and reducing ESG controversies. Their collaboration with management and shareholders can lead to more effective policies that enhance CSR goals and social performance of the firm. The results support the collective action framework where both management and workers get benefits of their collective actions towards improving the social performance of the organization [104]. This collaboration can help in revitalizing unions and mobilizing workers’ power towards achieving sustainable goals within the organization and the society at large.

However, it is imperative to mention that there are some limitations of the study. First, the study has uses data from Thomson Reuters Eikon which has limited data on certain variable; future research can also include other databases for improving the understanding of the important relationship between CSR and trade unions. Second, the trade union representation score only represents the percentage of employees who are members of trade union within the firm; however, it doesn’t entail their power and effectiveness in bargaining within the organization. The legal purview of the labour unions varies across the countries and therefore the effectiveness of trade unions. Future studies can focus on cross country analysis by means of qualitative interviews or primary data to gauge trade union power and effectiveness. Qualitative interviews can be conducted across different countries with union representatives and members to gauge their involvement in CSR and other corporate governance-related strategies. Such holistic approach can help in advancing stakeholder theory from the perspectives of IR. Third, the study has only focused on manufacturing sector due to the prevalence of trade union within it. However, future researchers can also involve other sectors to probe the suggested association between trade unions and CSR. As a new wave of unions in gig economy is rising where the notion of unions and the engagement of unions right from the formation of union is completely different from that of manufacturing. Future studies can also focus on effectiveness of unions in different sectors of economy to see their impact on CSR. Having said that, the results of the study present that trade unions are an important stakeholder in CSR and thus CSR-sustainability can be a positive step towards revitalising trade unions around the world [24].

## Supporting information

S1 Appendix (DOCX)

S2 Appendix (DOCX)
