# Supplementary material for: From union clout to corporate couture: Unveiling the impact of trade unions on corporate social responsibility
Source: PLoS One. 2025 Jan 9;20(1):e0311244. doi: 10.1371/journal.pone.0311244 (PMC11717276; doi:10.1371/journal.pone.0311244)
Supplement: S1 Appendix — (DOCX) [file pone.0311244.s001.docx]

**Appendix A**

| **Variables** | **Definitions from Thomson Reuters Terminal** (Refinitiv, 2023) |
| --- | --- |
| Trade union Representation score | Percentage of employees represented by independent trade union organizations or covered by collective bargaining agreements. - trade union representation percentage = number of employees represented by trade union members/total number of employees*100 - when both percentages of employees represented by trade union and percentage of employees covered by collective bargaining agreements are available, consider the percentage of employees covered by collective bargaining agreements |
| Management score | Management category score measures a company's commitment and effectiveness towards following best practice corporate governance principles. |
| Shareholders score | Shareholders category score measures a company's effectiveness towards equal treatment of shareholders and the use of anti-takeover devices. |
| ESG Controversies Score | ESG controversies category score measures a company's exposure to environmental, social and governance controversies and negative events reflected in global media. |
| CSR Sustainability Reporting Score | Does the company publish a separate CSR/H&S/Sustainability report or publish a section in its annual report on CSR/H&S/Sustainability? - any separate extra-financial report in which the company reports on the environmental and social impact of its operations - when the company publishes an extra financial report in a foreign language we answer as ‘True’ with a comment - web-based non-financial reports are also considered if data is updated yearly - integrated annual report with sustainability data is qualified information - CSR section from the annual report must consist of substantial data - exceptionally, if company report quantitative data exclusively in less than 5 pages can also be considered - CSR reports published bi-annually, current year when there is no report then data measure is answered ‘False’ - data only on community-focused report with community-related activities of the company, answer is ‘False’ |

**Reference**

Refinitiv. (2023). *Environmental, Social and Governance (ESG) Scores From Refinitiv*. https://www.lseg.com/en/data-analytics/sustainable-finance/esg-scores
