# Supplementary material for: From union clout to corporate couture: Unveiling the impact of trade unions on corporate social responsibility
Source: PLoS One. 2025 Jan 9;20(1):e0311244. doi: 10.1371/journal.pone.0311244 (PMC11717276; doi:10.1371/journal.pone.0311244)
Supplement: S2 Appendix — (DOCX) [file pone.0311244.s002.docx]

**Appendix B**

The appendix provides results for fixed effect panel data regression for unbalanced panel, partially balanced panel (panel with variable having information on 8 or more years) and partially balanced panel with imputed values. The results are presented below:

1. **Results for Unbalanced Panel with 2200 firms**

**Descriptive statistics**

| **Table 1** |  |  |
| --- | --- | --- |
| Distribution of firms across different manufacturing sectors | | |
| Industry | Number of entries | Percentage |
| Energy | 1,680 | 7.64 |
| Basic Materials | 2,890 | 13.14 |
| Industrials | 3,880 | 17.64 |
| Consumer cyclicals | 3,750 | 17.05 |
| Consumer non-cyclicals | 1,950 | 8.86 |
| Healthcare | 3,100 | 14.09 |
| Technology | 3,210 | 14.59 |
| Utilities | 1,540 | 7 |
| Total | 22,000 | 100 |

| **Table 2** |  |  |
| --- | --- | --- |
| Distribution of firms across different institutional categories | | |
| Institutional Category | Number of entries | Percentage |
| Collaborative Agglomerations | 150 | 0.69 |
| Coordinated Market Economy | 3,470 | 15.99 |
| Centralized tribe | 10 | 0.05 |
| Emergent Liberal Market Economy | 950 | 4.38 |
| Family led | 810 | 3.73 |
| Hierarchically Coordinated | 10 | 0.05 |
| Liberal Market Economy | 920 | 4.24 |
| State Led | 13,460 | 62.03 |
| Other | 1,920 | 8.85 |
| Total | 21700 | 100 |

| **Table 3** | | | | | |
| --- | --- | --- | --- | --- | --- |
| Summary statistics | | | | | |
| Variable | No. of observations | Mean | Std. Dev. | Min. | Max. |
| Trade Union Representation | 12,323 | 36.66593 | 34.95079 | 0 | 100 |
| Management Score | 14,912 | 54.57367 | 28.33218 | 0.031075 | 99.98057 |
| Shareholder Score | 14,912 | 51.53331 | 28.80391 | 0.050134 | 99.98057 |
| Log of Total Revenue | 20,539 | 21.30514 | 2.010536 | 6.907755 | 26.88825 |
| Total Revenue | 20,940 | 7.84E+09 | 2.08E+10 | 0 | 4.76E+11 |
| ESG Controversy Score | 14,912 | 89.55354 | 23.87206 | 0.704225 | 100 |
| CSR Reporting Score | 14,912 | 42.72142 | 35.64121 | 0 | 99.64539 |

| **Table 4** | | | | | | |  |
| --- | --- | --- | --- | --- | --- | --- | --- |
| Bivariate correlation between variables | | | | | | |  |
| Variables | (1) | (2) | (3) | (4) | (5) | (6) | (7) |
| (1) Trade Union Representation | 1.000 |  |  |  |  |  |  |
| (2) Management Score | 0.027** | 1.000 |  |  |  |  |  |
| (3) Shareholder Score | 0.009 | 0.132*** | 1.000 |  |  |  |  |
| (4) Log of Total Revenue | 0.196*** | 0.200*** | 0.032*** | 1.000 |  |  |  |
| (5) Total Revenue | 0.165*** | 0.133*** | -0.002 | 0.657*** | 1.000 |  |  |
| (6) ESG Controversy Score | -0.076*** | -0.074*** | -0.038*** | -0.343*** | -0.335*** | 1.000 |  |
| (7) CSR Reporting Score | 0.438*** | 0.212*** | 0.068*** | 0.350*** | 0.212*** | -0.161*** | 1.000 |

**** p<0.01, ** p<0.05, * p<0.1*

**Regression Results**

| **Table 5** | | | |
| --- | --- | --- | --- |
| Results of Regression Analysis for ESG Controversy (Sample of 2200 firms for 10 years) | | | |
|  | Model 1 | Model 2 | Model 3 |
| VARIABLES | Fixed Effect | Fixed Effect with control | Fixed Effect with country, industry, and category controlled (260 combinations) |
| *Independent Variables* |  |  |  |
| Trade union Representation | -0.063** | -0.059** | -0.105*** |
|  | (0.026) | (0.027) | (0.010) |
| Management score | 0.001 | -0.000 | -0.006 |
|  | (0.012) | (0.012) | (0.008) |
| Shareholder score | -0.005 | -0.006 | -0.012 |
|  | (0.012) | (0.012) | (0.007) |
| Year Effects | Yes | Yes | Yes |
| *Control Variable* |  |  |  |
| Log of Total Revenue |  | 0.299 | -4.501*** |
|  |  | (0.561) | (0.152) |
| Constant | 86.752*** | 80.038*** | 187.834*** |
|  | (1.412) | (12.291) | (3.172) |
|  |  |  |  |
| Observations | 12,323 | 12,173 | 12,012 |
| R-squared | 0.026 | 0.026 | 0.201 |
| Number of identifiers | 2,200 | 2,171 | 2,171 |

Note. *p < 0.1, **p < 0.05, ***p < 0.01. Standard errors in parentheses.

| **Table 6** | | | |
| --- | --- | --- | --- |
| Results of Regression Analysis for CSR Reporting (Sample of 2200 firms for 10 years) | | | |
|  | Model 1 | Model 2 | Model 3 |
| VARIABLES | Fixed Effect | Fixed Effect with control | Fixed Effect with country, industry, and category controlled (260 combinations) |
| *Independent Variables* |  |  |  |
| Trade union Representation | 0.064*** | 0.066*** | 0.194*** |
|  | (0.017) | (0.017) | (0.011) |
| Management score | 0.050*** | 0.050*** | 0.119*** |
|  | (0.008) | (0.008) | (0.009) |
| Shareholder score | 0.018** | 0.019** | 0.030*** |
|  | (0.007) | (0.008) | (0.008) |
| Year Effects | Yes | Yes | Yes |
| *Control Variable* |  |  |  |
| Log of Total Revenue |  | 0.622* | 6.882*** |
|  |  | (0.353) | (0.164) |
| Constant | 42.491*** | 29.229*** | -118.873*** |
|  | (0.888) | (7.745) | (3.428) |
|  |  |  |  |
| Observations | 12,323 | 12,173 | 12,012 |
| R-squared | 0.081 | 0.082 | 0.591 |
| Number of identifiers | 2,200 | 2,171 | 2171 |

Note. *p < 0.1, **p < 0.05, ***p < 0.01. Standard errors in parentheses.

1. **Results for Partially-balanced Panel (with data available on 8 or more years)**

**Descriptive statistics**

| **Table 1** |  |  |
| --- | --- | --- |
| Distribution of firms across different manufacturing sectors | | |
| Industry | Number of entries | Percentage |
| Energy | 690 | 10.21 |
| Basic Materials | 1300 | 19.23 |
| Industrials | 1,180 | 17.46 |
| Consumer cyclicals | 1,070 | 15.83 |
| Consumer non-cyclicals | 630 | 9.32 |
| Healthcare | 290 | 4.29 |
| Technology | 830 | 12.28 |
| Utilities | 770 | 11.39 |
| Total | 6,760 | 100 |

| **Table 2** |  |  |
| --- | --- | --- |
| Distribution of firms across different institutional categories | | |
| Institutional Category | Number of entries | Percentage |
| Collaborative Agglomerations | 60 | 0.9 |
| Coordinated Market Economy | 1,210 | 18.14 |
| Centralized tribe |  |  |
| Emergent Liberal Market Economy | 530 | 7.95 |
| Family led | 340 | 5.1 |
| Hierarchically Coordinated | 400 | 6 |
| Liberal Market Economy | 3,670 | 55.02 |
| State Led | 460 | 6.9 |
| Total | 6,670 | 100 |

| **Table 3** | | | | | |
| --- | --- | --- | --- | --- | --- |
| Summary statistics | | | | | |
| Variable | No. of observations | Mean | Std. Dev. | Min. | Max. |
| Trade Union Representation | 6,446 | 43.26818 | 33.05582 | 0 | 100 |
| Management Score | 6,688 | 60.44108 | 26.99557 | 0.458716 | 99.98057 |
| Shareholder Score | 6,688 | 53.214 | 28.60207 | 0.139276 | 99.97994 |
| Log of Total Revenue | 6,748 | 22.43157 | 1.351163 | 13.86525 | 26.88825 |
| Total Revenue | 6,748 | 1.39E+10 | 2.92E+10 | 1051000 | 4.76E+11 |
| ESG Controversy Score | 6,688 | 85.0129 | 27.40631 | 0.704225 | 100 |
| CSR Reporting Score | 6,688 | 54.72655 | 33.68892 | 0 | 99.63504 |

| **Table 4** | | | | | | |  |
| --- | --- | --- | --- | --- | --- | --- | --- |
| Bivariate correlation between variables | | | | | | |  |
| Variables | (1) | (2) | (3) | (4) | (5) | (6) | (7) |
| (1) Trade Union Representation | 1.000 |  |  |  |  |  |  |
| (2) Management Score | 0.027** | 1.000 |  |  |  |  |  |
| (3) Shareholder Score | 0.009 | 0.132*** | 1.000 |  |  |  |  |
| (4) Log of Total Revenue | 0.196*** | 0.200*** | 0.032*** | 1.000 |  |  |  |
| (5) Total Revenue | 0.165*** | 0.133*** | -0.002 | 0.657*** | 1.000 |  |  |
| (6) ESG Controversy Score | -0.076*** | -0.074*** | -0.038*** | -0.343*** | -0.335*** | 1.000 |  |
| (7) CSR Reporting Score | 0.438*** | 0.212*** | 0.068*** | 0.350*** | 0.212*** | -0.161*** | 1.000 |

**** p<0.01, ** p<0.05, * p<0.1*

**Regression Results**

| **Table 5** | | | |
| --- | --- | --- | --- |
| Results of Regression Analysis for ESG Controversy (Sample of 676 firms for 10 years) | | | |
|  | Model 1 | Model 2 | Model 3 |
| VARIABLES | Fixed Effect | Fixed Effect with control | Fixed Effect with country, industry, and category controlled (165 combinations) |
| *Independent Variables* |  |  |  |
| Trade union Representation | -0.105*** | -0.098** | -0.136*** |
|  | (0.038) | (0.038) | (0.016) |
| Management score | -0.002 | -0.002 | 0.008 |
|  | (0.017) | (0.017) | (0.013) |
| Shareholder score | 0.000 | -0.002 | -0.008 |
|  | (0.017) | (0.017) | (0.012) |
| Year Effects | Yes | Yes | Yes |
| *Control Variable* |  |  |  |
| Log of Total Revenue |  | 0.056 | -6.912*** |
|  |  | (1.013) | (0.309) |
| Constant | 82.648*** | 81.187*** | 239.577*** |
|  | (2.315) | (22.807) | (6.812) |
|  |  |  |  |
| Observations | 6,446 | 6,434 | 6,352 |
| R-squared | 0.026 | 0.026 | 0.230 |
| Number of identifiers | 676 | 675 | 675 |

Note. *p < 0.1, **p < 0.05, ***p < 0.01. Standard errors in parentheses.

| **Table 6** | | | |
| --- | --- | --- | --- |
| Results of Regression Analysis for CSR Reporting (Sample of 676 firms for 10 years) | | | |
|  | Model 1 | Model 2 | Model 3 |
| VARIABLES | Fixed Effect | Fixed Effect with control | Fixed Effect with country, industry, and category controlled (165 combinations) |
| *Independent Variables* |  |  |  |
| Trade union Representation | 0.069*** | 0.067*** | 0.235*** |
|  | (0.024) | (0.024) | (0.016) |
| Management score | 0.052*** | 0.049*** | 0.103*** |
|  | (0.010) | (0.010) | (0.013) |
| Shareholder score | 0.033*** | 0.032*** | 0.036*** |
|  | (0.010) | (0.010) | (0.012) |
| Year Effects | Yes | Yes | Yes |
| *Control Variable* |  |  |  |
| Log of Total Revenue |  | 2.042*** | 8.567*** |
|  |  | (0.622) | (0.302) |
| Constant | 53.710*** | 7.959 | -150.044*** |
|  | (1.418) | (14.004) | (6.651) |
|  |  |  |  |
| Observations | 6,446 | 6,434 | 6,352 |
| R-squared | 0.069 | 0.070 | 0.514 |
| Number of identifiers | 676 | 675 | 675 |

Note. *p < 0.1, **p < 0.05, ***p < 0.01. Standard errors in parentheses.

1. **Results for Partially-balanced Panel with Imputed values (with data available on 8 or more years)**

**Descriptive statistics:** The distribution of firms are same as partially-balanced panel.

| **Table 3** | | | | | |
| --- | --- | --- | --- | --- | --- |
| Summary statistics | | | | | |
| Variable | No. of observations | Mean | Std. Dev. | Min. | Max. |
| Trade Union Representation | 6,521 | 43.35723 | 33.03719 | 0 | 100 |
| Management Score | 6,689 | 60.43353 | 27.00061 | 0.458716 | 99.98057 |
| Shareholder Score | 6,689 | 53.20836 | 28.60365 | 0.139276 | 99.97994 |
| Log of Total Revenue | 6,749 | 22.43139 | 1.351145 | 13.86525 | 26.88825 |
| Total Revenue | 6,748 | 1.39E+10 | 2.92E+10 | 1051000 | 4.76E+11 |
| ESG Controversy Score | 6,689 | 85.01514 | 27.40487 | 0.704225 | 100 |
| CSR Reporting Score | 6,689 | 54.72967 | 33.68737 | 0 | 99.63504 |

| **Table 4** | | | | | | |  |
| --- | --- | --- | --- | --- | --- | --- | --- |
| Bivariate correlation between variables | | | | | | |  |
| Variables | (1) | (2) | (3) | (4) | (5) | (6) | (7) |
| (1) Trade Union Representation | 1.000 |  |  |  |  |  |  |
| (2) Management Score | 0.027** | 1.000 |  |  |  |  |  |
| (3) Shareholder Score | 0.012 | 0.133*** | 1.000 |  |  |  |  |
| (4) Log of Total Revenue | 0.195*** | 0.200*** | 0.032*** | 1.000 |  |  |  |
| (5) Total Revenue | 0.165*** | 0.133*** | -0.002 | 0.657*** | 1.000 |  |  |
| (6) ESG Controversy Score | -0.076*** | -0.074*** | -0.038*** | -0.342*** | -0.335*** | 1.000 |  |
| (7) CSR Reporting Score | 0.434*** | 0.212*** | 0.068*** | 0.350*** | 0.212*** | -0.161*** | 1.000 |

**** p<0.01, ** p<0.05, * p<0.1*

**Regression Results**

| **Table 5** | | | |
| --- | --- | --- | --- |
| Results of Regression Analysis for ESG Controversy (Sample of 676 firms for 10 years) | | | |
|  | Model 1 | Model 2 | Model 3 |
| VARIABLES | Fixed Effect | Fixed Effect with control | Fixed Effect with country, industry, and category controlled (165 combinations) |
| *Independent Variables* |  |  |  |
| Trade union Representation | -0.100*** | -0.093** | -0.134*** |
|  | (0.038) | (0.038) | (0.016) |
| Management score | 0.001 | 0.001 | 0.008 |
|  | (0.017) | (0.017) | (0.013) |
| Shareholder score | 0.001 | 0.001 | -0.008 |
|  | (0.017) | (0.017) | (0.012) |
| Year Effects | Yes | Yes | Yes |
| *Control Variable* |  |  |  |
| Log of Total Revenue |  | 0.059 | -6.876*** |
|  |  | (1.011) | (0.308) |
| Constant | 82.131*** | 80.463*** | 238.692*** |
|  | (2.306) | (22.753) | (6.778) |
|  |  |  |  |
| Observations | 6,521 | 6,510 | 6,427 |
| R-squared | 0.026 | 0.026 | 0.228 |
| Number of identifiers | 676 | 675 | 675 |

Note. *p < 0.1, **p < 0.05, ***p < 0.01. Standard errors in parentheses.

| **Table 6** | | | |
| --- | --- | --- | --- |
| Results of Regression Analysis for CSR Reporting (Sample of 676 firms for 10 years) | | | |
|  | Model 1 | Model 2 | Model 3 |
| VARIABLES | Fixed Effect | Fixed Effect with control | Fixed Effect with country, industry, and category controlled (165 combinations) |
| *Independent Variables* |  |  |  |
| Trade union Representation | 0.069*** | 0.066*** | 0.231*** |
|  | (0.023) | (0.023) | (0.016) |
| Management score | 0.053*** | 0.050*** | 0.105*** |
|  | (0.010) | (0.010) | (0.013) |
| Shareholder score | 0.034*** | 0.034*** | 0.035*** |
|  | (0.010) | (0.010) | (0.012) |
| Year Effects | Yes | Yes | Yes |
| *Control Variable* |  |  |  |
| Log of Total Revenue |  | 2.084*** | 8.643*** |
|  |  | (0.621) | (0.300) |
| Constant | 53.698*** | 6.961 | -151.550*** |
|  | (1.416) | (13.988) | (6.612) |
|  |  |  |  |
| Observations | 6,521 | 6,510 | 6,427 |
| R-squared | 0.068 | 0.070 | 0.513 |
| Number of identifiers | 676 | 675 | 676 |

Note. *p < 0.1, **p < 0.05, ***p < 0.01. Standard errors in parentheses.
